# Dissociation of behavioral and neural responses to provocation during reactive aggression in healthy adults with high versus low externalization

**DOI:** 10.3758/s13415-021-00981-y

**Published:** 2022-01-28

**Authors:** Julian Konzok, Gina-Isabelle Henze, Ludwig Kreuzpointner, Hannah L. Peter, Marina Giglberger, Christoph Bärtl, Claudia Massau, Christian Kärgel, Kathrin Weidacker, Boris Schiffer, Hedwig Eisenbarth, Stefan Wüst, Brigitte M. Kudielka

**Affiliations:** 1 Department of Medical Psychology, Psychological Diagnostics and Research Methodology, Institute of Psychology, University of Regensburg, Universitaetsstrasse 31, 93053 Regensburg, Germany; 2 Division of Forensic Psychiatry, LWL-University Hospital, Ruhr University Bochum, Bochum, Germany; 3 School of Psychology, University of Swansea, Swansea, UK; 4 School of Psychology, Victoria University of Wellington, Wellington, New Zealand

**Keywords:** Taylor Aggression Paradigm, Externalizing spectrum, fMRI, Anterior cingulate cortex

## Abstract

The externalizing spectrum describes a range of heterogeneous personality traits and behavioral patterns, primarily characterized by antisocial behavior, disinhibition, and substance (mis)use. In psychopathology, abnormalities in neural threat, reward responses and the impulse-control system may be responsible for these externalizing symptoms. Within the non-clinical range, mechanisms remain still unclear. In this fMRI-study, 61 healthy participants (31 men) from the higher versus lower range of the non-clinical variation in externalization (31 participants with high externalization) as assessed by the subscales disinhibition and meanness of the Triarchic-Psychopathy-Measure (TriPM) performed a monetary modified Taylor-Aggression-Paradigm (mTAP). This paradigm consisted of a mock competitive-reaction-time-task played against a fictional opponent with preprogrammed win- and lose-trials. In lose-trials, participants were provoked by subtraction of an amount of money between 0 and 90 cents. As a manipulation check, provocation induced a significant rise in behavioral aggression levels linked with an increased activation in the anterior cingulate cortex (ACC). High externalization predicted reduced ACC responses to provocation. However, high externalizing participants did not behave more aggressively than the low externalization group. Additionally, the high externalizing group showed a significantly lower positive affect while no group differences emerged for negative affect. In conclusion, high externalization in the non-clinical range was related to neural alterations in regions involved in affective decision-making as well as to changes in affect but did not lead to higher behavioral aggression levels in response to the mTAP. This is in line with previous findings suggesting that aberrations at multiple levels are essential for developing externalizing disorders.

**Supplementary Information:**

The online version contains supplementary material available at 10.3758/s13415-021-00981-y.

## Introduction

The externalizing spectrum model was originally developed for explaining the coincidence of a set of heterogeneous personality traits and behavioral patterns encompassing antisocial behavior, disinhibition, and substance (mis)use (Krueger et al., 2002; Krueger, Markon, Patrick, Benning, & Kramer, 2007; Patrick et al., 2013). However, empirical work has shown that externalization is a dimensional characteristic, distributed across the general population (Krueger, Markon, Patrick, Benning, & Kramer, 2007; Markon & Krueger, 2005). For example, the externalizing spectrum comprises variation in trait impulsivity as a personality characteristic as well as more extreme, and clinically-relevant, behavioral expressions which are indicative for, e.g., attention-deficit/hyperactivity disorder (ADHD), conduct disorder (CD), antisocial personality disorder (ASPD) and substance abuse disorders (SUD) (Zisner & Beauchaine, 2016). In particular, high trait impulsivity represents a risk factor for the development of externalizing disorders (Beauchaine, Zisner, & Sauder, 2017). Furthermore, individuals exhibiting high externalization often show emotional hyperreactivity as well as aggressive behavior (Bohnert, Crnic, & Lim, 2003; McLaughlin, Hatzenbuehler, Mennin, & Nolen-Hoeksema, 2011). However, most studies reporting increased aggressive behavior in the context of externalization were derived from clinical populations while evidence from the general population is still scarce (Brislin et al., 2019; White, Jarrett, & Ollendick, 2013b). According to the ontogenic process model of the externalizing spectrum by Beauchaine, Shader, and Hinshaw (2016), externalizing disorders are products of bidirectional transactions between individual vulnerabilities (e.g., impulsivity) and environments (e.g., maltreatment, neglect, reinforcement processes) over the life span. Hormonal and neural substrates of those processes comprise alterations in mesolimbic dopamine, hypothalamus-pituitary-adrenal (HPA) axis, prefrontal dopamine and amygdala function. Studying externalization within the non-clinical range and its corresponding biobehavioral substrates can lead to a better understanding of the processes derailing during the development of externalizing psychopathologies and provide support for this transactional perspective.

A well-established laboratory measure for behavioral aggression, in particular reactive aggression, is the Taylor Aggression Paradigm (TAP). In the original version by Taylor (1967), participants played a fictitious reaction time task against a mock opponent with default win and lose trials. In win trials, the participant was instructed to administer an electric shock to the mock opponent. Thereby, the selected intensities served as indicator for reactive aggression as provoked by respective punishment selections of the mock opponent in previous lose trials. Over the last decades, this paradigm was modified quantitatively (e.g., in terms of provocation, duration, number of trials) and qualitatively (e.g., kind of stimuli; Elson, Mohseni, Breuer, Scharkow, & Quandt, 2014). Meanwhile, a modified Taylor Aggression Paradigm (mTAP) was established using monetary stimuli (subtraction of money from a fictitious account) instead of electric shocks, noise or heat stimuli (Kogan-Goloborodko, Brügmann, Repple, Habel, & Clemens, 2016; Konzok et al., 2020; Wagels et al., 2018; Weidler et al., 2019). Recent research supports the validity of the monetarily modified TAP by showing a dose response effect of preceding provocation on the behavioral responses by the participant (Repple et al., 2017; Schneider et al., 2015) as well as by showing correlations with other measures of reactive aggression (e.g., self-reported aggression by questionnaire) (Konzok et al., 2020; Weidler et al., 2018). Furthermore, moderating effects of other factors known to influence reactive aggression (e.g., gender) was shown empirically (Konzok et al., 2020; Weidler et al., 2019). Finally, recent studies applied for the first time trial-by-trial analysis in order to account for individual aggression trajectories using multilevel approaches (Chester, 2019; Konzok et al., 2020; Wagels et al., 2018).

Investigating the neurobiology of reactive aggression, results from neuroimaging studies using mTAPs with noise, thermal or pneumatic stimuli indicated that retaliation is associated with altered activity in medial prefrontal cortex (mPFC), the orbitofrontal cortex (OFC), and superior temporal gyrus reflecting cognitive control processes (for review see Fanning, Keedy, Berman, Lee, & Coccaro, 2017). Increased provocation by the mock opponent induced greater activation in the amygdala, insula, anterior cingulate cortex (ACC), thalamus, and OFC (Buades-Rotger et al., 2016; Krämer, Jansma, Tempelmann, & Münte, 2007; Lotze, Veit, Anders, & Birbaumer, 2007). Particularly interesting are recent findings with the monetary mTAP showing that the mPFC, posterior parts of the superior and middle frontal gyrus as well as cingulate cortex (ACC, middle cingulate cortex), and insula are activated during the active selection of a punishment (reactive aggression). In contrast to the neural activity during reactive aggressive behavior, observing the subtraction of money from the own account by the mock opponent (provocation) was related to activity in the ACC, thalamus, nucleus caudatus, mPFC, and insula (Repple et al., 2017; Wagels et al., 2019; Weidler et al., 2018). In sum, findings from mTAP-studies with different types of stimuli suggest that the ACC, mPFC, and OFC play a key role in both, provocation processing and active aggression, while insula and amygdala activity rather reflects the experience of the current affective state during provocation.

Examining neural circuits mediating abnormal reactive aggression, the majority of studies focused on psychiatric disorders for which aggression is a main diagnostic criterion including externalizing disorders like ASPD and CD. These studies suggest that reactive aggressive behavior is associated with abnormalities in three neural systems implicated in the experience of aggression, decision making, and regulation of emotions (for review see Coccaro, Sripada, Yanowitch, & Phan, 2011).

Especially in externalizing disorders, neuroimaging studies revealed reduced neural activity during reward as well as emotion processing (including acute threat) and (poor) decision making in the OFC/ventromedial prefrontal cortex (vmPFC), ACC, striatum, amygdala, and insula as shown in patients with CD (for review see Fairchild et al., 2019), ADHD (Plichta et al., 2009), SUD (Koob & Volkow, 2010) and antisocial behavioral tendencies (Oberlin et al., 2012). However, findings are less consistent regarding the direction of amygdala abnormalities. Patients with CD showed slightly a blunted amygdala response during emotion processing or in response to visual threat cues (Hwang et al., 2016; Sterzer, Stadler, Krebs, Kleinschmidt, & Poustka, 2005), while results in ADHD patients are divergent (Marsh et al., 2008; Plichta et al., 2009; Sterzer et al., 2005). Additionally, in antisocial tendencies, amygdala reactivity is moderated by callous-unemotional traits (Viding, Fontaine, & McCrory, 2012). These dysfunctions are hypothesized to enhance the probability for impulsive behavior and reactive aggression (Blair, Veroude, & Buitelaar, 2018). Furthermore, in a social exchange paradigm, individuals exhibiting disruptive behavior problems and low callous-unemotional traits are associated with reduced OFC activity during retaliation and blunted amygdala-OFC-connectivity during high provocation compared to healthy controls (White et al., 2016). From a developmental perspective, the ontogenic process model of the externalizing spectrum (Beauchaine, Shader, & Hinshaw, 2016) assumes that externalizing disorders share common vulnerabilities (e.g., dysregulation in prefrontal areas, amygdala, mesolimbic system). However, the individual expression depends strongly on the environment and protective factors.

With this, the question arises whether externalization in the non-clinical normal range might also be related to altered neural processing of provocation and reactive aggression. To this point, evidence from the general population is still scarce. One study revealed aberrant activity in threat and reward systems in response to (un)pleasant pictures associated with trait disinhibition within a healthy sample. However, the authors did not explicitly control for psychiatric disorders (Foell et al., 2016). Additionally, activation in the nucleus caudatus, dorsomedial prefrontal cortex, anterior insula, ACC and periaqueductal gray was positively correlated with retaliation during the above-mentioned social exchange paradigm in healthy subjects, which was partly modulated by callous-unemotional traits. In the OFC/vmPFC and posterior cingulate cortex, the authors found a negative association between neural activation and punishment levels (White, Brislin, Meffert, Sinclair, & Blair, 2013a; White, Brislin, Sinclair, & Blair, 2014).

### Research Questions

Our main research aim was to investigate behavioral, affective, and neural processes mediating reactive aggression in a non-clinical, healthy sample with high versus low externalization.

First, we expected a dose-response effect of provocation on behavioral reactive aggression in the monetary mTAP accompanied by altered neural responses in the ACC as well as OFC/vmPFC during retaliation. Limbic and mesolimbic systems including amygdala, insula, and ACC should be involved during higher levels of provocation, according to previous findings from mTAP-studies and models of reactive aggression (Fanning et al., 2017).

In participants scoring high on externalization, we assumed higher aggression levels, especially after higher levels of provocation. Furthermore, we presumed enhanced negative affect as well as reduced positive affect in high compared to low externalizing participants in response to the monetary mTAP.

According to findings from externalizing pathologies and the ontogenic process model of the externalizing spectrum, healthy participants with high compared to low externalization were expected to manifest reduced activation in inhibitory control areas (ACC, OFC) during active aggressive behavior and altered (meso)limbic activity while being provoked by the opponent as well as in response to the outcome (win vs. lose).

## Methods

### Participants

Sixty-three preselected subjects (age: *M* = 23.62, *SD* = 3.81, range 18 – 34; 31 men, 32 women) from the higher (*n* = 32) versus lower (*n* = 31) range of the normal variation in externalization were tested twice in the magnetic resonance imaging (MRI) scanner. The assignment to one of the two externalization groups was based on the scores in the TriPM (Patrick, 2010; Patrick, Fowles, & Krueger, 2009) within the scopes of the highest (*Q*_75_) or lowest quartile (*Q*_25_) of the subscales disinhibition (*Q*_75_ = 36, *Q*_25_ = 29) and meanness (*Q*_75_ = 33, *Q*_25_ = 26) as derived from a large online assessment in the general population (Eisenbarth, Castellino, Alpers, Kirsch, & Flor, 2012). Volunteers scoring high on the personality trait psychopathy were not eligible, i.e., volunteers scoring within the upper quartile of the TriPM subscale boldness (*Q*_75_ = 55) were not selected.

All participants reported to be free of acute and chronic illness, mental, and psychiatric disorders as assessed with the one-hour lasting German version of the Structured Clinical Interview for DSM-IV Axis I Disorders (SCID-I) and Axis II Personality Disorders (SCID; Wittchen, Zaudig, & Fydrich, 1997), criminal history, current use of drugs and medication with glucocorticoids as well as MRI-scanner contraindications. Owing to poor image acquisition and missing saliva samples, two subjects were excluded from functional MRI (fMRI) analyses rendering a final sample of *N* = 61. All subjects gave written informed consent and were compensated with 100 € or course credits. This experiment was approved by the ethics committee of the University of Regensburg.

### Procedure

The current research project consisted of two fMRI sessions on two different days. At the first scanning session, neural stress responses were investigated applying the Scan*STRESS* paradigm (reported in Konzok et al., 2021). The second scanning session (reported here) comprised the monetary mTAP. Participants arrived at least 30 min before the beginning of the scanning session (starting after 12 p.m.). In order to increase the credibility of the cover story, subjects were introduced to a confederate of the same gender and informed that they will act as opponents in a competitive reaction time task while being in adjacent scanner rooms. After a monetary mTAP training session consisting of five trials, participants were transferred into the scanner room and passed a resting state (RS, 18 min) and diffusion tensor imaging (DTI, 22 min) sequence (results not presented in the present manuscript). Subsequently, we conducted the monetary mTAP (see below). After the scanning session, participants filled in an in-house deception check questionnaire to identify participants who expressed suspicion regarding the cover story (see Konzok et al., 2020).

During the experimental session, saliva was collected by Cortisol Salivettes (Sarstedt, Nümbrecht, Germany) at eight time points: -75 min (shortly after arrival), -55 min (directly before transfer into scanner), -1 min before the start of the monetary mTAP as well as +1 min, +10 min, +20 min, +30 min, and +45 min (C1 – C8) thereafter. At each saliva sampling point, subjects completed the state version of the Positive and Negative Affect Schedule (PANAS; Watson, Clark, & Tellegen, 1988) or answered it per hand signal while lying in the scanner.

One week after the scanning session, participants received a link to an online assessment including a trait aggression questionnaire (“Kurzfragebogen zur Erfassung von Aggressivitätsfaktoren” (K-FAF; Heubrock & Petermann, 2008), the Buss Perry Aggression Questionnaire (BPAQ; Buss & Perry, 1992), the Brown-Goodwin Lifetime History of Aggression Scale (BGHA; Brown, Goodwin, Ballenger, Goyer, & Major, 1979), and the Reactive Proactive Questionnaire (RPQ; Raine et al., 2006).

### Monetary modified Taylor Aggression Paradigm (mTAP)

**Fig. 1:**
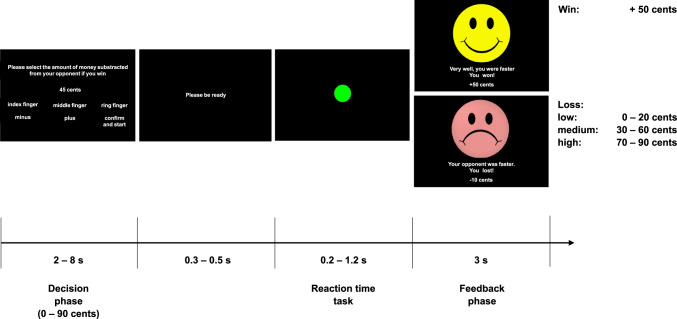
Procedure of a single trial in the monetary mTAP. Reprinted from “Validation of a monetary Taylor Aggression Paradigm: Associations with trait aggression and role of provocation sequence,” by J. Konzok, L. Kreuzpointner, G.-I. Henze, L. Wagels, C. Kärgel, K. Weidacker, B. Schiffer, H. Eisenbarth, S. Wüst, and B. M. Kudielka, 2020, Journal of Experimental Social Psychology, 88, 103960. (original in German, translated for publication)

The monetary mTAP is a mock competitive reaction time task with a fictional opponent. Each trial consists of a decision phase, the reaction time task itself and a feedback phase. At the beginning of each trial (decision phase), the participant has to set a stake between 0 and 90 euro cents on the computer with a given setting start point of 45 cents for each trial. The setting start point cannot be chosen as final stake. Following the recommendations by Tedeschi and Felson (1994), the amount of 0 cents is included as nonaggressive option (see also Elson et al., 2014). For the reaction time task, participants are instructed to press a button as quickly as possible as soon as a green circle appears on the computer screen. After reaction times slower than 500 ms, the feedback: “You haven’t pressed a button. Please react as quickly as possible.” is presented. In case the participant presses the button before the green circle appears the trial is repeated. It is emphasized that the selected amount will be subtracted from the opponent in case the opponent loses the reaction time task. If the participant loses the trial, the punishment level selected by the fictional opponent will be delivered to the participant (feedback phase). In lose trials, the level of provocation is defined by the amount of subtracted money by the opponent. In case of winning, the participant always receives 50 cents (see Fig. 1). While performing the task, participants do not receive any feedback of their current account balance (see also Konzok et al., 2020).

Participants performed 100 randomly presented trials, with preprogrammed 40 win and 60 lose trials. The unprovoked first trial was omitted from the analyses.

### Materials, Biochemical Analysis, and Data Acquisition

The monetary mTAP was presented on a monitor via a stimulus computer using the software Presentation (Version 19.0; Neurobehavioral Systems, San Francisco, CA, USA). After test sessions, saliva samples were stored at -20°C. Analyses were performed by the biochemical laboratory at the University of Trier, Germany. Cortisol was assayed in duplicate using a time-resolved immunoassay with fluorometric detection (DELFIA). Inter- and intra-assay coefficients of variation were below 10 %, respectively.

To collect fMRI data, we used a MAGNETOM Siemens 3T Prisma scanner (Siemens AG; Erlangen, Germany) and a 64-channel head coil. Between scalp and head coil foam paddings were positioned to prevent extensive movement. A T2*-weighted echo-planar imaging (EPI) sequence (TR = 2 000 ms; TE = 30 ms, flip angle = 90°, FOV = 192 x 192 mm^2^, 64 x 64 matrix, 37 slices with 1-mm gap, slice thickness = 3.0 mm, voxel size = 3 x 3 x 3 mm^3^, interleaved) was used to create functional scans and a T1-weighted magnetization-prepara high-pass filter correction of 128ed rapid gradient-echo (MP-RAGE) sequence (TR = 2400 ms, TE = 2.18 ms, flip angle = 9°, voxel size = 0.8 x 0.8 x 0.8 mm^3^, distance factor: 50%) for structural scans.

### Psychometric Measures

To define the two quasi-experimental groups, we used the three subscales boldness, meanness and disinhibition of the TriPM (Patrick, 2010; Patrick et al., 2009) (see above). The four-point answering format ranges from 1 = *not true at all* to 4 = *completely true*. The PANAS (Watson et al., 1988) is composed of 20 items and measures positive and negative affect on a five-point answering scale. Trait aggression was evaluated by the German K-FAF (Heubrock & Petermann, 2008) comprising 49 items pooled to five subscales (spontaneous aggression, reactive aggression, excitability, aggression inhibition and self-aggression) and the 28-item BPAQ (Buss & Perry, 1992), which includes four subscales (physical aggression, verbal aggression, anger, and hostility) with a five-point answering format (1 = *not true at all* to 5 = *completely true*). The BGHA (Brown et al., 1979) measures aggressive behavior by eleven questions to be answered on a four-point rating scale across three developmental stages of life (childhood, adolescence and adulthood). The 23-item RPQ (Raine et al., 2006) consists of two subscales with a three-point answering scale, namely proactive and reactive aggression.

### Statistical Analysis

To assess potential differences between the two externalization groups, independent Welch-test comparisons regarding demographic variables and aggression questionnaires were performed using R (version 3.5.1; R Core Team, 2018) with the packages afex (Singmann, Bolker, Westfall, Aust, & Ben-Shachar, 2020), lme4 (Bates, Mächler, Bolker, & Walker, 2014), lmerTest (Kuznetsova, Brockhoff, & Christensen, 2017), psych (Revelle, 2019) and sjstats (Lüdecke, 2020). Repeated measures analyses of variance (ANOVAs, Greenhouse–Geisser corrected) were performed for salivary cortisol (‘time’ [8 cortisol samples] x ‘gender’ [women, men] x ‘externalization’ [high, low]).

According to an already validated evaluation strategy of the monetary mTAP with linear mixed models (LMM; Konzok et al., 2020), we analyzed participants' aggression in response to the amount of money subtracted by the mock opponent in the previous trial (provocation) and added a random intercept by subject accounting for interindividual variability as well as a random slope for provocation by subject. Due to a better use of all available information (conditional *R*^*2*^, the variance explained by the entire model), a continuous provocation variable (0 – 90 cents) was preferred over a categorized variable provocation (low [0 – 20 cents] vs. medium [30 – 60 cents] vs. high [70 – 90 cents]) consisting of two estimates (with low provocation serving as the reference standard to which the effects of medium and high provocation were related to) which had been applied earlier (Konzok et al., 2020) (see Supplementary Table 1 and 2 for detailed information). In a next step, the predictors of interest, externalization (low vs. high) and trait reactive aggression assessed by the K-FAF were included. Additionally, the model was enhanced by the factors gender as sole factor, an interaction term provocation by gender, and deception check ensuring correct variance allocations.

To evaluate the goodness of fit regarding the random and fixed effect structure, we built four different models. Model 1 only consisted of a random intercept for participant (null-model); model 2 included a fixed effect for ‘provocation’; in model 3 and 4 a random slope for provocation by participant was added; in addition, model 4 (full model) contained also fixed effects for the predictors of interest and additional factors. All fixed effects were tested with an *F*-Tests type III using a Satterthwaite approximation of the degrees of freedom (Kenward & Roger, 1997).

A repeated measures analysis of variance (rmANOVA) was performed for positive and negative affect (‘time’ [8 assessments] x ‘externalization’ [low, high]). For this analysis, eight participants had to be removed because of missing time points.

### Functional MRI data analysis.

Imaging analysis was performed with SPM12 (Wellcome Department of Imaging Neuroscience, University College London, London, UK). Data preprocessing started with the realignment by registering the scans to the mean images. Afterwards, slice timing was conducted with the first slice as reference. Then, functional mean images and the T1-weighted scan were co-registered. In the context of segmentation and normalization, functional and structural images were transformed into the standard space defined by the Montreal Neurological Institute (MNI). At the end of the preprocessing, we smoothed the functional images with an isotropic Gaussian kernel of 8 mm full-width-at-half-maximum. Additionally, a high-pass filter correction of 128 s was arranged removing low-frequency drifts.

#### Whole brain analysis

For each single subject (first level analysis), a general linear model (GLM) was fitted with the four regressors of interest (decision phase after high and low provocation, feedback phase with high and low provocation) as well as eleven regressors of no interest (decision phase after winning trials as well as winning feedback phase, the reaction time task, the six realignment parameters). To enhance statistical power, decision phase after medium provocation and feedback phase with medium provocation were also excluded from the analyses as regressors of no interest.

On the second level, we performed four factor-analysis of variance (full factorial design) for decision and feedback phase, respectively one model in response to provocation (provoked trials only: preceding trial was lost) and one model as function of outcome (preceding winning and losing trial). The four models included the factors previous provocation (low vs. high), respectively previous outcome (won vs. lost) and externalization (low vs. high). All whole brain analyses were conducted with family-wise error (FWE: *p* < .05) correction based on Gaussian random field theory. *F*-contrasts for main effect of ‘provocation’ respectively outcome and externalization as well as the interaction effects were conducted for decision and feedback phase.

#### ROI analysis

For *post-hoc* region of interest (ROI) analyses (as derived from the ontogenic process model as well as earlier empirical results), masks in the ACC, OFC, amygdala, insula, nucleus accumbens, nucleus caudatus, and putamen were created using the SPM Anatomy toolbox (Eickhoff et al., 2005). Average beta estimates were extracted employing the MarsBaR toolbox for SPM (http://marsbar.sourceforge.net/). The parameter estimates entered rmANOVAs, Bonferroni corrected at *p* = .05.

#### Parametric modulation

To explore activations in brain regions covarying with the amount of money selected by the participants during decision phases, an aggression response-related blood oxygen level dependent (BOLD) analysis was conducted by building a second first level model for each participant. Further, differing decision phases in terms of the outcome of the previous trial (win > lost), this model included a parametric modulator for the amount of money subtracted by the participants in each trial. On the second level, we performed a two-sample *t*-test regarding the parametric modulator after losing trials (reactive aggression modulated neural response). Additionally, high and low externalizing participants were contrasted regarding the aggression response-related activation pattern (high > low, low > high). Due to missing variance in aggressive response selection (*SD* = 0), five participants had to be removed from these analyses (*N* = 56). For this explorative analysis, an original threshold was set at *p* = .001 and FWE was corrected on cluster level at *p* = .05. For anatomy localization, the SPM Anatomy toolbox (Eickhoff et al., 2005) was used. *Post-hoc* ROI analyses were conducted within the significant cluster from the previous contrast.

## Results

### Descriptive Results

Consistent with phenotypic characteristic of externalization, the high externalization group showed significant greater values in the disinhibition and meanness scales of the TriPM compared to the low externalization group. Significant group differences also emerged in four out of five scales of the K-FAF (except aggression inhibition), the five scales of the BPAQ, the two scales of the RPQ and the adolescence scale of the BGHA (see Table 1). The aggression inhibition subscale of the K-FAF showed that low externalizing participants scored significantly higher. In supplementary two-factors ANOVAs, we additionally analyzed potentially ‘gender’ and ‘externalization by gender’ effects showing that none of the questionnaire scores differed between men and women (all *F* < 3.34 all *p* > .073). The deception check questionnaire revealed that 21 participants (33%) reported at least some suspicion regarding the cover story.

**Table 1:** Mean ± SD of demographic, behavioral, and psychometric variables and results of welch tests and Cohen’s d contrasting the two externalization groups

		High externalization		Low externalization		*t*	*df*	*d*	*p*-value
Age in yrs.		Men (*n* = 16)	Women (*n* = 16)	Men (*n* = 15)	Women (*n* = 16)	1.05	60.93	.298	-0.27
TriPM									
	Disinhibition	40.25 (± 4.57)	42.87 (± 5.88)	27.07 (± 1.62)	26.94 (± 2.05)	-14.56	38.34	.001*	3.68
	Meanness	40.31 (± 5.45)	38.88 (± 4.03)	23.20 (± 1.86)	22.87 (± 1.82)	-18.31	40.04	.001*	4.63
	Boldness	47.75 (± 5.52)	47.75 (± 5.51)	51.53 (± 2.97)	49.06 (± 3.96)	2.41	57.37	.019	-0.61
K-FAF									
	Spontaneous aggression	17.13 (± 8.89)	20.38 (± 7.63)	7.20 (± 6.21)	3.88 (± 2.55)	-7.74	50.57	.001*	1.97
	Reactive aggression	27.44 (± 8.97)	28.38 (± 8.39)	15.80 (± 7.15)	11.69 (± 4.69)	-7.55	56.81	.001*	1.92
	Excitability	20.19 (± 10.20)	24.38 (± 8.19)	11.33 (± 6.99)	11.88 (± 5.73)	-5.34	54.35	.001*	1.36
	Self-aggression	18.06 (± 10.12)	23.88 (± 9.45)	8.87 (± 5.37)	8.25 (± 5.27)	-6.17	46.95	.001*	1.56
	Inhibition	17.94 (± 5.99)	16.44 (± 5.19)	20.66 (± 4.89)	21.81 (± 4.10)	3.21	58.97	.002*	-0.82
BPAQ									
	Physical aggression	20.50 (± 6.65)	21.00 (± 8.18)	14.87 (± 2.50)	11.50 (± 2.58)	-5.42	41.54	.001*	1.37
	Verbal aggression	13.50 (± 3.16)	14.25 (± 4.40)	11.07 (± 2.46)	10.75 (± 1.61)	-3.89	47.87	.001*	0.99
	Anger	18.31 (± 4.76)	20.38 (± 5.98)	13.80 (± 4.06)	12.88 (± 2.96)	-5.25	53.31	.001*	1.34
	Hostility	19.81 (± 6.63)	22.13 (± 6.13)	14.20 (± 5.16)	13.13 (± 4.51)	-5.16	57.41	.001*	1.32
	Sum	72.13 (± 14.34)	77.75 (± 21.28)	53.93 (± 9.03)	48.25 (± 8.11)	-6.70	45.52	.001*	1.70
BGHA									
	Childhood	17.31 (± 5.10)	14.63 (± 2.83)	14.60 (± 5.11)	12.13 (± 1.86)	-2.56	60.84	.013	0.65
	Adolescence	20.19 (± 4.02)	18.38 (± 5.06)	15.20 (± 4.23)	13.94 (± 1.81)	-4.75	55.70	.001*	1.21
	Adulthood	15.38 (± 3.56)	13.63 (± 2.22)	13.07 (± 2.22)	12.38 (± 2.19)	-2.68	56.37	.010	0.68
RPQ									
	Proactive aggression	13.13 (± 1.45)	14.00 (± 2.48)	12.27 (± 0.46)	12.19 (± 0.40)	-3.61	33.75	.001*	0.91
	Reactive aggression	16.94 (± 4.12)	18.75 (± 3.70)	14.47 (± 3.00)	14.56 (± 1.50)	-4.09	50.17	.001*	1.04

Notes: yrs. = years. * comparison survived Bonferroni correction at *p* < .05 (adjusted *p*-value = .0025).

For salivary cortisol responses, we found a significant main effect of ‘time’ (*F*(2.30,133.12) = 25,61, *p* < .001, η_p_^2^ = .31) indicating a slight anticipatory cortisol increase after arrival (from -75 to -55 min) but decreasing levels after the transfer into the scanner throughout the rest of the session (including the mTAP) reflecting the normal circadian rhythm. A significant main effect of ‘gender’ (*F*(1,58) = 5.90, *p* = .018, η_p_^2^ = .09) as well as interaction ‘time by gender’ (*F*(2.30,133.12) = 4,63, *p* = .008, η_p_^2^ = .07) indicated a more pronounced anticipatory response in men while cortisol levels were comparable between men and women during the mTAP session. The main effect of ‘externalization’ as well as none of the other interaction effects reached significance (*F*s < 1.20, *p*s > .309, η_p_^2^ < .02).

### Behavioral and Affective Responses

Within the context of model building for behavioral aggression levels, inclusion of provocation as fixed effect (model 1 vs. model 2; ∆χ^2^(*df* = 1) = 143.44, *p* < .001, ∆*BIC* = 135.23) and as random effect (model 2 vs. model 3; ∆χ^2^(*df* = 2) = 196.04, *p* < .001, ∆*BIC* = 179.58) resulted in an improved model fit using likelihood ratio tests, indicating a significant impact of provocation on behavioral responses allover (fixed effect) as well as on individual level (random effect). Comparing model 3 and model 4 including all predictors as fixed effects, we observed no improvement of the goodness fit (model 3 vs. model 4; ∆χ^2^(*df* = 6) = 12.18, *p* = .440, ∆*BIC* = -37.15). (see Supplementary Table 1 for detailed model comparison).

**Fig. 2:**
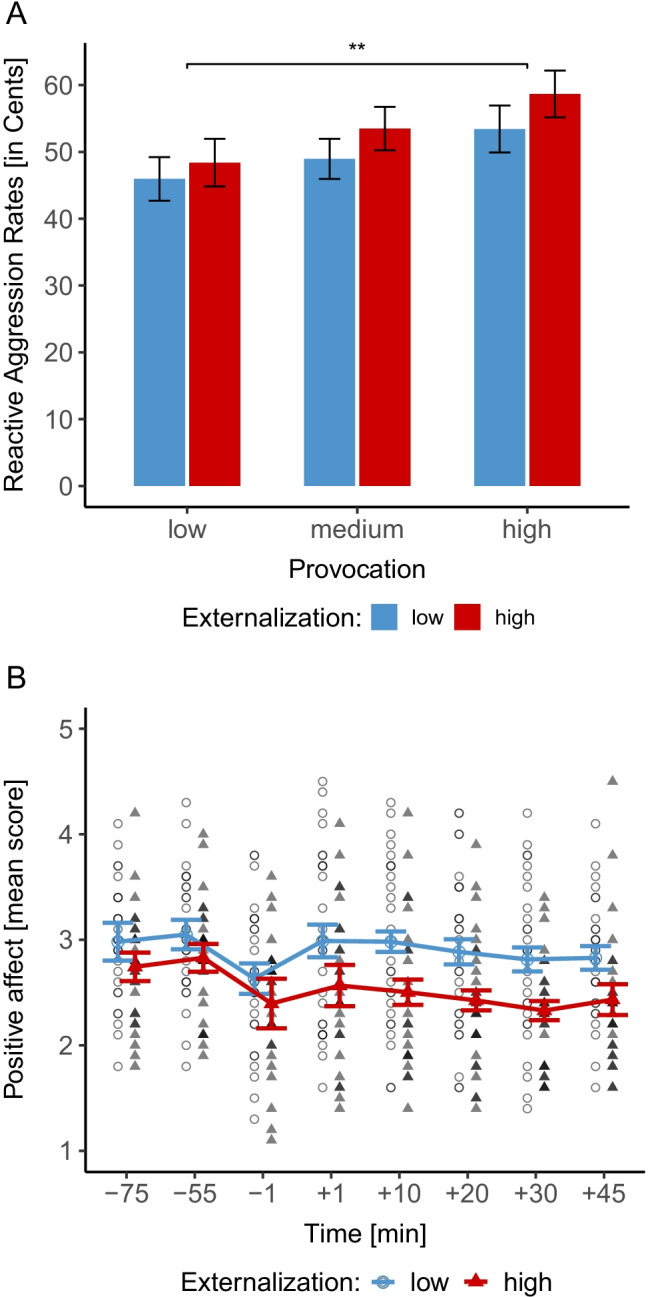
Mean (± SEM) aggression levels in response to the provocation of the fictional opponent (categorized provocation variable presented for illustrative reasons, see Supplementary Table 1 und 2 for detailed information), separated for the low and high externalization group (**A**). PANAS scores for positive affect (**B**)

Evaluating fixed effects of model 4, Table 2 depicts *t*-tests for individual fixed-effect estimates and *F*-tests (Satterthwaite approximation of the degrees of freedom) for overall effects of fixed factors. *F*-tests confirmed a main effect of ‘provocation’, though no main effect ‘externalization’ nor interaction of provocation and externalization (see Fig. 2). Additionally, there was no significant interaction ‘provocation by gender’ (see Table 2).

**Table 2:** Parameter estimates and F-tests for overall effects for the model (model 4) with reactive aggression as dependent variable in the monetary mTAP

	beta	SEM	*t*	*p*-value	*df*_*n*_*/df*_*d*_	*F*	*p*-value	η_p_^2^
Intercept	49.09	6.60	7.44	**< .001**				
Provocation	0.13	0.05	2.89	**.006**	1/47.31	24.26	< .001	.34
Externalization (0=low, 1=high)	-5.64	8.58	-0.66	.516	1/32.16	0.43	.516	.01
Provocation x externalization	0.04	0.05	0.81	.421	1/47.31	0.66	.421	.01
Gender	3.85	6.64	0.58	.566	1/28.07	0.34	.566	.01
Provocation x gender	-0.05	0.05	-0.97	.339	1/47.31	0.93	.339	.02
Deception check (0=no suspicion, 1=suspicion)	-6.81	6.03	-1.13	.267	1/31.57	1.27	.268	.04
K-FAF trait reactive aggression (normalized)	5.61	4.01	1.40	.171	1/31.56	1.96	.171	.06

Note: *df*_*n*_ = degrees of freedom numerator, *df*_*d*_ = degrees of freedom denominator.

The additional factors gender, the interaction provocation by gender, deception check and *z*-normalized trait reactive aggression assessed by the K-FAF manifested no predictive value for the aggression levels in the laboratory paradigm. Since externalization and trait reactive aggression appeared to be no independent factors (see Table 1), we subsequently computed the variance inflation factor (*VIF*) showing low correlations with other predictors (*VIF* < 5) for externalization (*VIF* = 2.26) and *z*-normalized trait reactive aggression (*VIF* = 1.95).

PANAS scores for positive affect showed a significant decline over time (*F*(3.74, 198.37) = 8.78, *p* < .001, η_p_^2^ = .14) and a significant ‘externalization’ effect (*F*(1, 53) = 5.70, *p* = .021, η_p_^2^ = .10) exhibiting a higher positive affective state in the low externalization group (see Fig. 2B) but no interaction effect (*F*(3.74, 198.37) = 1.39, *p* = .240, η_p_^2^ = .03). For negative affect, we found a significant ‘time’ effect (*F*(4.26, 225.55) = 10.88, *p* < .001, η_p_^2^ = .17) indicating a decrease over the experimental session, however, no group differences (*F*(1, 53) = 0.11, *p* = .744, η_p_^2^ = .00) nor interaction effect (*F*(4.26, 225.55) = 1.18, *p* = .319, η_p_^2^ = .02) emerged.

### Neural Results

#### Decision phase

Based on provoked trials (i.e., the preceding trial was lost), whole brain results for the decision phase (reflecting behavioral reactive aggression) revealed no suprathreshold cluster (*k* > 20) for the main effects of ‘provocation’ and ‘externalization’ nor for the interaction provocation by externalization.

**Fig. 3:**
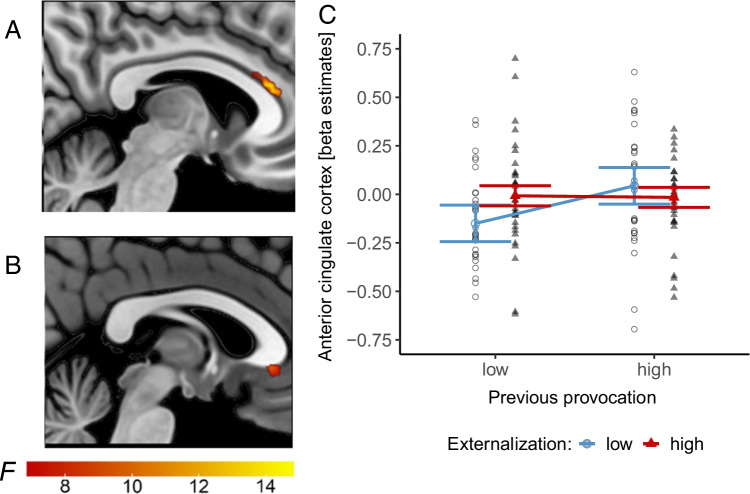
Provocation (low vs. high) x externalization (low vs. high) ROI analysis during decision phase. Location of the activation peaks for the ‘provocation’ effect in the pregenual region of the rACC (**A**). Activation peaks for the provocation by externalization interaction in the subgenual region of the rACC (**B**). Results of the ROI analyses for the provocation by externalization interaction in the bilateral ACC (**C**)

Subsequent ROI analyses with repeated measure ANOVAs revealed a significant effect of the previous provocation (low vs. high) on beta estimates and the interaction provocation by externalization only in the rostral part of the ACC during the decision phase, surviving Bonferroni correction for multiple comparisons (adjusted *p* = .025). Activation peaks for this ‘provocation’ effect are located in the pregenual region and for the interaction effect in the subgenual region of the rostral ACC (rACC; see Fig. 3A and B). The paired comparison of bilateral ACC beta estimates after high and low provocation showed a significant difference in the low externalization group (*t*(29) = -2.98, *p* = .006), but not in the high externalization group (*t*(32) = 0.23, *p* = .820). Moreover, results in bilateral ACC beta estimates after the high compared to the low provocation condition (*M*_*high prov*_ – *M*_*low prov*_) differed significantly between externalization groups, indicating higher differences in the low externalization group (*M*_*high ex*_ = -0.01, *M*_*low ex*_ = 0.19, *t*(45.34) = 2.72, *p* = .009). Therefore, increased activation in the rACC after high compared to low provocation during the decision phase was greater in the low than high externalization group (see Fig. 3B and C). Thus, the high externalization group showed reduced neural responses of the ACC to provocation compared to the low externalization group. For detailed information and statistics, we refer to Table 3.

**Table 3:** Results of ROI analyses during decision and feedback phases

Phase	Region	Provocation effect			Externalization effect			Interaction provocation x externalization		
		*F*	*p*-value	η_p_^2^	*F*	*p*-value	η_p_^2^	*F*	*p*-value	η_p_^2^
Decision										
	ACC	6.46	.014*	.10	0.35	.554	.01	7.65	.008*	.11
	OFC/vmPFC	2.79	.100	.04	0.00	.986	< .01	4.60	.036	.07
Feedback										
	ACC	0.03	.853	.00	0.97	.328	.02	0.00	.973	< .01
	Insula	4.73	.034	.07	2.11	.151	.03	0.04	.834	<. 01
	Amygdala	1.45	.233	.02	1.45	.234	.02	0.05	.826	< .01

Notes: ACC = anterior cingulate cortex, OFC = orbitofrontal cortex, vmPFC = ventromedial prefrontal cortex, * comparison survived Bonferroni correction at *p* < .05.

Analyzing the decision phase depending on the outcome of the previous trial (F-contrast), we found a significant main effect of previous ‘outcome’ in one suprathreshold cluster including the right posterior cingulate cortex (*F* = 38.05; x = 9, y = -37, z = 11). This indicates more activation after win compared to lose trials as shown by a *post-hoc t*-contrast (previous outcome: won > lost). No ‘externalization’ effect nor interaction of ‘externalization and outcome’ could be found in whole brain analyses.

#### Parametric modulation of aggression response-related activation

The BOLD response within the left precentral gyrus, left superior temporal gyrus, and the right nucleus caudatus covaried positively with the amount of money selected by the participants during decisions that followed lose trials (reactive aggression) (see Supplementary Table 3). There was no difference in aggression response-related activation between the low and high externalization group nor between men and women.

#### Feedback phase

Based on provocation trials (i.e., the preceding trial was lost), there was no ‘externalization’ effect nor interaction of externalization and provocation for the feedback phase on whole brain level. Within ROI analyses, the ‘provocation’ effect reached significance only in the insula, but did not survive Bonferroni correction (adjusted *p* = .016). Besides this, neural activation was neither affected by externalization nor the provocation by externalization interaction in the amygdala, insula, and ACC. For detailed information and statistics see Table 3.

Analyzing the feedback phase depending on the outcome of the previous trial (won > lost), three suprathreshold clusters with a peak maximum in the right (*F* = 83.04; x = 12, y = -82, z = 29) and left (*F* = 71.06; x = 0, y = -82, z = 26) cuneus, right nucleus caudatus (*F* = 143.18; x = 15, y = 8, z = -10), right (*F* = 41.84; x = 33, y = -13, z = -1) and left (*F* = 120.19; x = -12, y = 8, z = -10) putamen, and left rACC (*F* = 50.10; x = 0, y = 35, z = 11) were rendered by factorial analysis for the main effect of ‘outcome’. *Post-hoc t*-contrast (won > lost) yielded an activation pattern in these areas. Externalization solely and the interaction outcome by externalization did not reveal suprathreshold clusters. *Post-hoc* ROI analyses did not show significant ‘externalization’ or interaction effects in the nucleus accumbens (*F*s < 0.29, *p*s > .592), nucleus caudatus (*F*s < 1.13, *p*s > .292), and putamen (*F*s < 1.92, *p*s > .167) during feedback.

#### Deception check

To check for potentially influencing effects of the variable deception check, we performed the above referred ROI analyses without participants (*n* = 21, 33%) who expressed suspicion regarding the cover story. During the decision phase, ROI analysis revealed a marginal significant ‘provocation’ effect in the bilateral ACC (*F*(1, 39) = 3.79, *p* = .059, η_p_^2^ = .09). The interaction provocation by externalization in the ACC remained significant (*F*(1, 39) = 4.52, *p* = .040, η_p_^2^ = .10).

During the feedback phase, a marginal significant ‘provocation’ effect on insula activation could be found (*F*(1, 39) = 4.02, *p* = .052, η_p_^2^ = .09).

## Discussion

The current study investigated behavioral, affective, and neural processes mediating reactive aggression in externalization in the non-clinical range. In line with previous results, the participants of our study responded on average more aggressively after higher levels of provocation in the monetary mTAP. On a neural level, this effect was associated with the ACC (activation peak in the pregenual part of the rACC) showing more activation during aggressive responses after high compared to low provocation in ROI analyses. This is in line with most of previous studies using the mTAP with either noise (Beyer, Münte, Erdmann, & Krämer, 2014; Krämer et al., 2007; Krämer, Riba, Richter, & Münte, 2011) or monetary stimuli (Repple et al., 2017). While the caudal or dorsal ACC is linked to cognitive processes, the rACC is involved in emotional processing (for review see Etkin, Egner, & Kalisch, 2011; Stevens, Hurley, & Taber, 2011). In particular, the activation in the rACC is associated with negative emotions such as anger and disgust (Dougherty et al., 1999; Phillips, Drevets, Rauch, & Lane, 2003).

Additionally, in the current study, the BOLD response in the nucleus caudatus covaried significantly with the amount of money selected by the participants after lose trials (reactive aggression). This is in accordance with the majority of studies applying different versions of the mTAP (Beyer et al., 2014; Gan, Sterzer, Marxen, Zimmermann, & Smolka, 2015; Krämer et al., 2007; Lotze et al., 2007) showing an association between activity of the nucleus caudatus and reactive aggression levels. Interestingly, the nucleus caudatus is also known to be involved in decisions or actions that are motivated by anticipation of rewards (e.g., O'Doherty et al., 2004). This applies also for reward processing during social interactions. For instance, during an economic exchange paradigm, the nucleus caudatus played a key role in altruistic punishment (De Quervain, Fischbacher, Treyer, & Schellhammer, 2004) and the authors concluded that this reflects expected satisfaction from punishment as reward.

Unexpectedly, we found neither an ‘externalization’ effect nor a significant impact of the interaction provocation by externalization for aggression levels in this monetary mTAP. This was astonishing, given that our healthy participants with higher externalization scores showed significantly higher self-reported trait aggression (K-FAF, BPAQ, RPQ), with comparable high effect sizes for proactive and reactive aggression scales (see Table 1). Results from previous studies showed a positive correlation between the scores of the TriPM subscales and self-report aggression questionnaires in a community sample, with proactive aggression subscales correlating more strongly with the TriPM than reactive aggression subscales (van Dongen, Drislane, Nijman, Soe-Agnie, & van Marle, 2017). Thus, the current data support the view that participants with high externalizing within a non-clinical range indeed describe themselves as more aggressive in a self-report questionnaire. However, within the scope of a laboratory behavioral aggression task presented as competitive reaction time task, no differences in behavioral aggression levels between high and low externalizing participants could be found.

Contrary to our predictions, we found a moderating effect of externalization only on positive affective states, but not on negative affect. However, results from a recent affective dynamics study in females pointed to a similar pattern showing association between externalizing disorders and less persistent positive affect as well as more variable positive emotionality (Scott et al., 2020). Thus, our results in healthy participants are consistent with Scott et al.’s conclusion that positive emotions are more transient in externalizing disorders. Further, it can be speculated that this finding might, to a lesser extent, reflect sensitivity to emotionally rewarding and punishing cues as described in clinically-relevant externalization (Scott et al., 2020).

In the current study, healthy high externalizing participants showed reduced responses to provocation in the ACC (activation peak in the subgenual region of the rACC) compared to the low externalization group. Findings from studies using EEG indicate that high externalization in healthy subjects is associated with a reduced amplitude of the error-related negativity (ERN) brain response, reflecting reduced self-monitoring (Hall, Bernat, & Patrick, 2007; Patrick et al., 2006). Interestingly, brain imaging studies using source analyses suggest that the ERN is primarily generated by the ACC (Dehaene, Posner, & Tucker, 1994; Miltner et al., 2003). Additionally, earlier studies revealed that reduced activation in the subgenual portion of the rACC is associated with alterations in emotion regulation (Etkin & Wager, 2007). Moreover, in affective decision making, blunted ACC activation was associated with reduced emotional control in CD patients (Cappadocia, Desrocher, Pepler, & Schroeder, 2009; Stadler et al., 2007). Consequently, within the non-clinical range the reduced rACC responses in high externalizing participants may reflect the variations in regulating emotional responses, respectively emotional sensitivity.

Thus, high externalization predicts reduced neural responses to provocation in the ACC; however, externalization has no predictive value for behavioral aggression levels during the mTAP. As suggested by the dual-system model (Beauchaine et al., 2017), two neurobiological systems are involved in promoting externalizing behavior across the lifespan: the emotional circuit localized in mesolimbic and limbic areas as well as the control circuit (e.g., PFC/ ACC). It is hypothesized that the normal developmental delay of the control compared to the emotional system is responsible for a period of increased vulnerability to externalizing problems and can lead to poor affective decision making as well as unfavorable reward and sensation seeking. However, in our study comprised of healthy subjects, higher externalization was not associated with aberrant neural processes in the emotional circuit including reward-related areas during provocation or reactive aggression. Thus, it can be speculated that alterations in one of these circuits might lead to manifestations of externalization only within the non-clinical range while deficits in both circuits enhance the likelihood for developing externalizing behavior within the psychopathological range. In line with this reasoning, Gordon, Baird, and End (2004) also yielded no behavioral group differences during an affective recognition task in non-clinical participants scoring high on psychopathy, but observed some susceptibility in the emotion circuit. In support of these supposed associations between behavioral problems and the degree of neural abnormalities, fMRI studies with externalizing disorders demonstrated an increase in neural activity in the mesolimbic system and an improvement of clinical symptoms after the administration of the dopamine agonist methylphenidate in patients with ADHD (Vles et al., 2003). Additionally, reduced frontal brain activation in an inhibitory task predicted later alcohol problems in pre-symptomatic adolescents (Norman et al., 2011). Likewise, mesolimbic dopamine dysfunction has been identified as neural correlate of trait impulsivity conferring vulnerability to externalizing pathologies (see Gatzke-Kopp, 2011). In order to clarify these associations, future work should focus on externalization in clinical and non-clinical samples applying experimental paradigms that are able to activate these neurobiological systems differentially.

Regarding the deception check, at the behavioral level, suspicion about the cover story did not lead to different aggression responses in the laboratory paradigm. At least, from a statistical point of view, suspicion about our cover story had no significant influence on the behavioral and neural outcome. Moreover, neural results could most widely be confirmed in the subsample of participants reporting no suspicion.

At this point, some limitations of our study have to be mentioned. First, prior to the monetary mTAP, participants went through a RS and DTI sequence (to be reported elsewhere). Although these sequences demanded no specific task performance and did not evoke specific affective states conferring to monetary mTAP performance, a nonsystematic effect of fatigue cannot be excluded. Second, although our sample size is suitable for a fMRI study, larger sample sizes enhancing statistical power might be needed to detect group differences in the monetary mTAP. Third, in the current paper an extreme group approach within the healthy (nonclinical) range was applied including participants scoring high versus low in the respective subscales of the TriPM. This design was realized in order to reach sufficient statistical test power. It should be noted, though, that with this approach, information about the intermediate range could not be collected. However, given the early stage of research in this field, the chosen approach appears reasonable (Preacher, 2014; Preacher, Rucker, MacCallum, & Nicewander, 2005). Finally, we mainly included university students potentially limiting the generalizability to other populations within the non-clinical range.

## Conclusions

We successfully induced provocation dependent aggressive behavior linked with increased activation in the ACC. Although high externalizing participants did not behave more aggressively compared to the low externalization group, aberrant activation in the ACC could be observed even within this non-clinical, healthy, range. Based on these findings, it might be speculated that additional dysfunctional regulation in other circuits that mediate pathological externalizing symptoms (e.g., in the emotional circuit), are essential for developing externalizing disorders like ADHD, CD and SUD.

## Supplementary Information

ESM 1 (DOCX 57 kb)
