# Supplementary material for: Dissociation of behavioral and neural responses to provocation during reactive aggression in healthy adults with high versus low externalization
Source: Cogn Affect Behav Neurosci. 2022 Jan 28;22(5):1130–44. doi: 10.3758/s13415-021-00981-y (PMC9458579; doi:10.3758/s13415-021-00981-y)
Supplement: Supplementary file 1 — (DOCX 57 kb) [file 13415_2021_981_MOESM1_ESM.docx]

**SUPPLEMENTS: Dissociation of behavioral and neural responses to provocation during reactive aggression in healthy adults with high versus low externalization**

Julian Konzok ^1^, Gina-Isabelle Henze ^1^, Ludwig Kreuzpointner ^1^, Hannah Peter ^1^, Marina Giglberger ^1^, Christoph Bärtl ^1^, Claudia Massau ^2^, Christian Kärgel ^2^, Kathrin Weidacker ^3^, Boris Schiffer ^2^, Hedwig Eisenbarth ^4^, Stefan Wüst ^1^ and Brigitte M. Kudielka ^1^

^1^ Institute of Psychology, University of Regensburg, Germany.

^2^ Division of Forensic Psychiatry, LWL-University Hospital, Ruhr University Bochum, Germany.

^3^ School of Psychology, University of Swansea, Swansea, Wales, United Kingdom.

^4^ School of Psychology, Victoria University of Wellington, New Zealand.

Correspondence concerning this article should be addressed to:

Julian Konzok, Department of Medical Psychology, Psychological Diagnostics and Research Methodology, University of Regensburg, Universitaetsstrasse 31, 93053 Regensburg, Germany.

E-mail: julian.konzok@ur.de

## Supplementary Table 1

*Four linear mixed models with reactive aggression as dependent variable in the monetary mTAP (continuous provocation variable)*

|  | Model 1 | Model 2 | Model 3 | Model 4 |
| --- | --- | --- | --- | --- |
| Intercept | **51.49 *****  (2.41) | **45.67 *****  (2.45) | **45.70 *****  (2.63) | **49.09 *****  (6.60) |
| Provocation (continuous) |  | **0.13 *****  (0.01) | **0.13 *****  (0.02) | **0.13 ****  (0.05) |
| Externalization (0=low, 1=high) |  |  |  | -5,64  (8.58) |
| Provocation x externalization |  |  |  | 0.04  (0.05) |
| Gender |  |  |  | 3.85  (6.64) |
| Provocation x gender |  |  |  | -0.05  (0.05) |
| Deception check (0=no suspicion, 1=suspicion) |  |  |  | -6.81  (6.03) |
| K-FAF trait reactive aggression (normalized) |  |  |  | 5.61  (4.01) |
| ICC: participant | .49 |  |  |  |
| AIC | 32 865.14 | 32 723.69 | 32 531.66 | 32 531.48 |
| BIC | 32 883.81 | 32 748.58 | 32 569.00 | 32 606.15 |
| Log Likelihood | -16 429.57 | -16 357.85 | -16 259.83 | -16 253.74 |
| Num. obs. | 3723 | 3723 | 3723 | 3723 |
| Num. groups: participant | 63 | 63 | 63 | 63 |
| Var: participant (Intercept) | 358.87 | 359.1 | 418.39 | 661.48 |
| Var: residual | 372.66 | 357.72 | 328.65 | 326.84 |
| Var: participant (Intercept) provocation |  |  | 0.03 | 0.04 |
| Cov: participant (Intercept) |  |  | -1.32 | -2.41 |
| Marginal *R^2^* |  | .02 | .02 | .06 |
| Conditional *R^2^* | .49 | .51 | .55 | .65 |

Notes. Model 1 only consists of a random intercept for participant (null-model); model 2 includes a fixed effect for provocation; in model 3 and 4 a random slope for provocation by participant is added; in addition, model 4 (full model) contains also fixed effects for externalization, gender, deception check and the trait reactive aggression scale of the K-FAF; *** *p* < .001, ** *p*<.01, * *p* < .05.

## Supplementary Table 2

*Four linear mixed models with reactive aggression as dependent variable in the monetary mTAP (**categorical provocation variable)*

|  | Model 1 | Model 2 | Model 3 | Model 4 |
| --- | --- | --- | --- | --- |
| Intercept | **51.35 *****  (2.44) | **46.97 *****  (2.48) | **47.00 *****  (2.58) | **51.37*****  (5.35) |
| Provocation (medium) |  | **4.18 *****  (0.77) | **4.15 *****  (1.10) | 3.31  (1.94) |
| Provocation (high) |  | **9.02 *****  (0.77) | **8.99 *****  (1.81) | **8.81****  (3.19) |
| Externalization (0=low, 1=high) |  |  |  | -7.00  (7.27) |
| Provocation (medium) x externalization |  |  |  | 2.20  (2.22) |
| Provocation (high) x externalization |  |  |  | 3.00  (3.65) |
| Gender |  |  |  | 2.05 (5.28) |
| Provocation (medium) x gender |  |  |  | -0.52  (2.22) |
| Provocation (high) x gender |  |  |  | -2.65  (3.65) |
| Deception check (0=no suspicion, 1=suspicion) |  |  |  | -5.35  (5.07) |
| K-FAF trait reactive aggression (normalized) |  |  |  | 6.36  (3.53) |
| ICC: participant | .49 |  |  |  |
| AIC | 32 865.14 | 32 733.42 | 32 557.62 | 32 534.41 |
| BIC | 32 883.81 | 32 764.53 | 32 619.85 | 32 646.41 |
| Log Likelihood | -16 429.57 | -16 361.71 | -16 268.81 | -16 249.2 |
| Num. obs. | 3723 | 3723 | 3723 | 3723 |
| Num. groups: participant | 63 | 63 | 63 | 63 |
| Var: participant (Intercept) | 358.87 | 359.05 | 393.1 | 404.22 |
| Var: residual | 372.66 | 359.51 | 331.54 | 331.62 |
| Var: provocation (medium) |  |  | 40.79 | 41.65 |
| Var: provocation (high) |  |  | 167.55 | 169.99 |
| Cov: participant (Intercept) provocation (medium) |  |  | -44.54 | -53.29 |
| Cov: participant (Intercept) provocation (high) |  |  | -67.69 | -80.57 |
| Cov: participant (Intercept) provocation (medium and high) |  |  | 82.32 | 83.63 |
| Marginal *R^2^* |  | .02 | .02 | .06 |
| Conditional *R^2^* | .49 | .50 | .55 | .56 |

Notes. Model 1 only consists of a random intercept for participant (null-model); model 2 includes a fixed effect for provocation; in model 3 and 4 a random slope for provocation by participant is added; in addition, model 4 (full model) contains also fixed effects for externalization, gender, deception check and the trait reactive aggression scale of the K-FAF; *** *p* < .001, ** *p*<.01, * *p* < .05.

## Supplementary Table 3

Activation peaks within a significant cluster covarying positively with selected aggression levels (parametric modulation)

| **Cluster**  **size (k)** | **Cytoarchitectonic location** | ***T*** | **x** | **y** | **z** |
| --- | --- | --- | --- | --- | --- |
| 217 | N/A | 4.43 | -27 | -22 | 53 |
|  | L Precentral Gyrus | 4.35 | -30 | -28 | 62 |
|  | L Precentral Gyrus | 4.21 | -39 | -25 | 65 |
|  | L Precentral Gyrus | 4.20 | -33 | -19 | 68 |
| 201 | L Superior Temporal Gyrus | 4.34 | -51 | -28 | 17 |
|  | N/A | 4.25 | -33 | -37 | 26 |
|  | N/A | 4.22 | -39 | -40 | 23 |
|  | L Rolandic Operculum | 3.97 | -45 | -28 | 23 |
|  | N/A | 3.86 | -36 | -13 | 29 |
|  | N/A | 3.73 | -42 | -10 | 29 |
| 110 | R Nucleus Caudatus | 5.26 | 18 | 17 | 17 |
|  | N/A | 4.96 | -3 | -4 | 20 |
|  | N/A | 4.61 | -9 | -10 | 29 |
|  | N/A | 4.35 | -12 | 8 | 26 |
|  | N/A | 3.81 | 3 | 14 | 8 |
|  | N/A | 3.77 | 6 | 20 | 11 |
|  | N/A | 3.7 | 12 | 2 | 26 |
|  | N/A | 3.63 | 6 | 2 | 20 |

Notes: L = left, R = right. N/A = Not found in any probability map.
